# The Hygiene of Children's Hair

**Published:** 1909-09-25

**Authors:** 


### Public Health and Hygiene.

### THE HYGIENE OF CHILDREN'S HAIR.

The School Medical Officer of the Willesden Local Education Authority recently issued a circular letter to the parents of children attending public elementary schools which appears to have excited a good deal of comment, and, it is said, not a little indignation. The circular letter reads as follows:—

Dear Sir or Madam,—As a result of the medical inspection of school-children, it has been discovered that a very large number of the children are sent to school in a verminous condition. To those careful and scrupulous parents who keep their children clean and free from this disgraceful state this must be a source of great annoyance and constant trouble, since their own children are thus necessarily exposed in school to verminous contamination.

An effort is now being made to free our public elementary schools from these pests, and I am making this appeal to all parents sending their children to such schools to assist me in the endeavour to protect their children from becoming verminous. Many parents desirous of keeping their children's heads clean find it almost impossible to do so in the case of girls, on account of the fact that they wear long hair. There is no need for young girls to wear their hair long. The hair would ultimately grow stronger if it were kept close-cut until the child was 10 or 12 years of age. If the cleanly parents will set the example of letting their girls wear short hair while at school much more hygienic conditions will be established in our public schools, and the prevention of vermin, ringworm, and other parasitic conditions of the head greatly facilitated. On the eve of the holidays I venture to make this appeal to parents to send all children to school with short hair.

There should at least be no objection to doing this in the case of the younger children attending the infants' departments, and there can be no doubt if this is done that long hair in schoolgirls will come to be recognised as the badge of uncleanliness.

But the sensible people who recognise the claims of hygiene must set the example to those parents who are insensible of the shame which should be felt at the evidence of parental neglect presented in the verminous and dirty state of a child's head.

Yours faithfully,
William Butler, School Medical Officer.

The *Daily News*, commenting upon the circular, considers that its issue displays on the part of the school medical officer concerned, "a remarkable lack of tact," and "sympathises with the mothers of Willesden in their determination to disregard the suggestion of the medical officer."

We venture on our part to extend our sympathy to a medical officer who in his efforts to raise the hygienic standard of the schools for which he is responsible, has to encounter not only the prejudices of parents, clean and unclean, and the direct antagonism of the great unwashed, but the abuse also of certain organs of the daily Press.

It is so frequently the duty of public servants to say and do the unpalatable things which are necessary for the common good that to charge them with "remarkable lack of tact" when they deliberately undertake an unpopular course is unworthy of a journal which claims to voice the spirit of reform.

And where does the *Daily News* go for its facts, or does it trouble about such important matters at all when it refers to the objectionable state of things which inspired the circular as "existing doubtless in but a few cases"? The sooner it is recognised that our public elementary schools are in a disgracefully verminous condition, the better it will be for that large section of the public which prefers the pseudo-æsthetic satisfaction of long hair to a sound hygienic taste in clean heads.

We probably underestimate when we say that more than half the girls attending schools maintained out of the rates and taxes are verminous. It is true that boys are not free from the affliction, but the fact has been established that they do not suffer to anything like the same extent as girls, and the reason lies in the fashion which dictates that boys shall wear their hair short. Those who have had most experience in dealing with the verminous condition of school children's hair are satisfied that only by a change in this fashion can a radical and permanent improvement be effected. Many working-class mothers have not time to give to long hair the attention it requires if it is to be kept clean, and once nits have been deposited on the hair, the prolonged exclusion from school necessary, if only vermin free children are to be admitted, encroaches most seriously upon the school-time of the child. In many schools if the system of rigid exclusion of verminous children were religiously carried out, the girls' departments would have repeatedly to be entirely closed. The plain facts are that thousands of cleanly parents, highly sensitive to the disgrace which properly is felt in harbouring vermin, are compelled to send their children to schools which are infested with pediculi.

It is no longer possible to close our eyes to the fact that our public elementary schools are in this respect a disgrace to those responsible for its continuance. If a remedy is to be found in the uniform practice among children of wearing short hair, the price does not seem exorbitant, and parents seriously desirous of the hygienic welfare of their children will do well to adopt a practice which, as the School Medical Officer of Willesden clearly sees, will in time cause the present vogue to be looked upon as "the badge of uncleanliness."

We are pleased to be in a position to affirm that, notwithstanding statements to the contrary in the daily press, the issue of the circular has effected a material improvement in the condition of Willesden scholars. Many parents, we understand, have acted upon the advice tendered by the Medical Officer, while the parents of children who have been persistently verminous have had less compunction in resorting to a form of treatment deprived of the stigma attaching to a measure ostensibly designed not to prevent but to remedy the condition. It is a curious feature in the social life of our time and a main difficulty in the attainment of the hygienic life that people who do not object to being in fact verminous in person and dirty in their habits or environment are yet most sensitive to the imputation that they are so.

